# Spatial analyses of *Plasmodium knowlesi* vectors with reference to control interventions in Malaysia

**DOI:** 10.1186/s13071-023-05984-x

**Published:** 2023-10-09

**Authors:** Sandthya Pramasivan, Romano Ngui, Nantha Kumar Jeyaprakasam, Van Lun Low, Jonathan Wee Kent Liew, Indra Vythilingam

**Affiliations:** 1 Department of Parasitology, Faculty of Medicine, Universiti Malaya (UM), Kuala Lumpur, Malaysia; 2 Department of ParaClinical Sciences, Faculty of Medicine and Health Sciences, Universiti Malaysia Sarawak (UNIMAS), Sarawak, Malaysia; 3 Biomedical Science Program, Center for Toxicology and Health Risk Studies, Faculty of Health Sciences, Universiti Kebangsaan Malaysia, Kuala Lumpur, Malaysia; 4 Tropical Infectious Diseases Research & Education Centre (TIDREC), Universiti Malaya (UM), Kuala Lumpur, Malaysia; 5 Environmental Health Institute, National Environment Agency, Singapore, Singapore

**Keywords:** *Anopheles*, Distribution, Leucosphyrus group, Predictive vector map, Malaysia

## Abstract

**Background:**

Malaria parasites such as *Plasmodium knowlesi*, *P. inui*, and *P. cynomolgi* are spread from macaques to humans through the Leucosphyrus Group of *Anopheles* mosquitoes. It is crucial to know the distribution of these vectors to implement effective control measures for malaria elimination. *Plasmodium knowlesi* is the most predominant zoonotic malaria parasite infecting humans in Malaysia.

**Methods:**

Vector data from various sources were used to create distribution maps from 1957 to 2021. A predictive statistical model utilizing logistic regression was developed using significant environmental factors. Interpolation maps were created using the inverse distance weighted (IDW) method and overlaid with the corresponding environmental variables.

**Results:**

Based on the IDW analysis, high vector abundances were found in the southwestern part of Sarawak, the northern region of Pahang and the northwestern part of Sabah. However, most parts of Johor, Sabah, Perlis, Penang, Kelantan and Terengganu had low vector abundance. The accuracy test indicated that the model predicted sampling and non-sampling areas with 75.3% overall accuracy. The selected environmental variables were entered into the regression model based on their significant values. In addition to the presence of water bodies, elevation, temperature, forest loss and forest cover were included in the final model since these were significantly correlated. *Anopheles* mosquitoes were mainly distributed in Peninsular Malaysia (Titiwangsa range, central and northern parts), Sabah (Kudat, West Coast, Interior and Tawau division) and Sarawak (Kapit, Miri, and Limbang). The predicted *Anopheles* mosquito density was lower in the southern part of Peninsular Malaysia, the Sandakan Division of Sabah and the western region of Sarawak.

**Conclusion:**

The study offers insight into the distribution of the Leucosphyrus Group of *Anopheles* mosquitoes in Malaysia. Additionally, the accompanying predictive vector map correlates well with cases of* P. knowlesi* malaria. This research is crucial in informing and supporting future efforts by healthcare professionals to develop effective malaria control interventions.

**Graphical Abstract:**

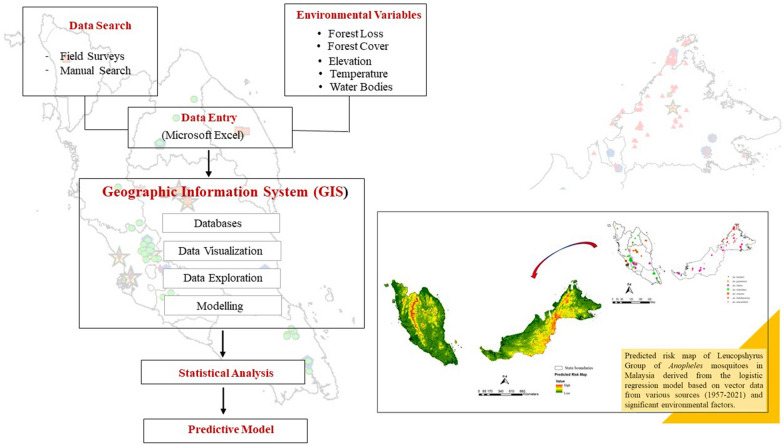

**Supplementary Information:**

The online version contains supplementary material available at 10.1186/s13071-023-05984-x.

## Background

Malaria is a significant global health concern that continues to cause fatalities and illnesses, especially in tropical areas [1]. The abundance of mosquito vectors, which thrive in suitable environments, such as those characterized by high humidity, precipitation, temperature and climate seasonality, contributes to the high incidence rate of malaria [2]. Although synthetic insecticides have been utilized to control these vectors, the growing resistance of malaria vectors to chemicals poses a threat to malaria prevention [2, 3]. Therefore, there is a need for more effective control measures to address this issue.

Although *Plasmodium knowlesi* has become more prevalent in recent years, the emergence of *Plasmodium cynomolgi* and *Plasmodium inui* as zoonotic malaria in Southeast Asia has complicated measures aimed at malaria elimination [4–6]. The WHO reports that countries will not achieve malaria elimination status if the number of *P. knowlesi* cases remains high [7]. Studies have identified several mosquito species from the Leucosphyrus Group of the genus *Anopheles*, including *An. cracens* [8, 9], *An. latens* [10], *An. balabacensis* [11, 12], and *An. introlatus* [13], that can transmit *P. knowlesi* to humans.

Most strategies for controlling malaria have focused on eliminating the disease in humans. This is understandable since humans account for the majority of malaria cases worldwide. However, the idea that simian malaria would rarely spill over into humans, proposed in the 1960s, is now outdated [14]. Recent developments have shown that zoonotic malaria is now a significant public health concern in Southeast Asia. Therefore, before declaring malaria eradication status, it is crucial to reconsider the threat of simian malaria and establish monitoring and control strategies [15–18]. Hence, mapping the distribution of simian malaria vectors in Southeast Asia is essential to the implementation of effective surveillance and control measures to eliminate the disease.

Comprehending the spatial and temporal pattern of simian malaria vectors is crucial, as it helps identify hotspot areas for vector abundance and allocating resources. Many studies have effectively used global geospatial techniques in mosquito environmental research [19–22]. These tools have also helped predict habitat suitability, which aids in designing optimal mosquito vector control strategies based on precise spatial and temporal information databases [23–25]. Geospatial mapping has the potential to identify larval habitats covering a large geographic area, which may be difficult or impossible to obtain through field surveys [20, 26].

Due to deforestation and changes in land use, certain *Anopheles* species from the Leucosphyrus Group have become more prevalent in farms and villages [27, 28]. However, there is still a need for high-quality knowledge on the distribution of these vectors throughout Malaysia. The spatial distribution of simian malaria vectors is crucial to determining effective vector control strategies but, unfortunately, there is currently a lack of information on their distribution throughout the country. Understanding the transmission patterns and geographical distribution of simian malaria parasites in Malaysia is essential for developing efficient disease control strategies and identifying how ecologies affect the risks of simian malaria. Therefore, this study aims to create a geographical distribution map and a predictive risk map based on the ecology of specific vectors of *P. knowlesi*. This information will enable possible interventions that can be used for vector control.

## Methods

### Data search

**Fig. 1:**
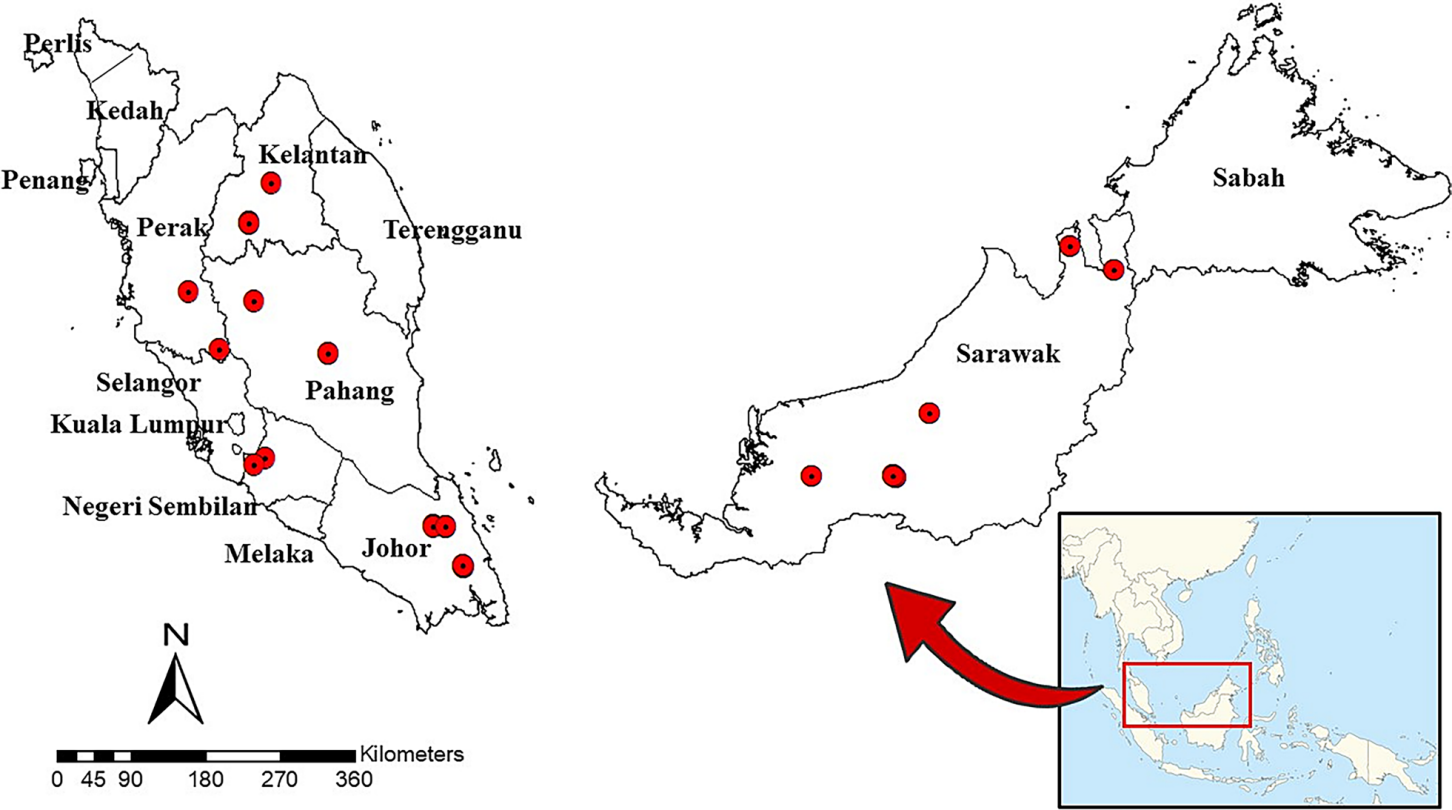
Location of collection sites of *Anopheles* mosquitoes in Malaysia

Relevant information on the Leucosphyrus Group of *Anopheles* mosquitoes was obtained through a combination of: (i) an extensive search of published articles on the *Anopheles leucosphyrus* sensu lato (*A. leucosphyrus* s.l.) mosquitoes between 1957 and 2021; (ii) mosquito sample collections carried out in the course of the present study from June 2019 until January 2021 in Malaysia (Fig. 1), the details of which regarding the sample collection are described in a previous study [29]; and (iii) direct contacts with district officers for *Anopheles* collection sites coordinates or unpublished research data were used for the spatial analysis. Online platform databases, such as PubMed, Medline and Google Scholar, were searched used to identify relevant studies on distribution of the simian malaria vector. We also compiled information from gray literature, such as hard copies of old publications, reports, thesis and dissertations pertinent to the research.

### Geo-positioning procedures

The surveyed geographic coordinates were determined using electronic resources, including GeoNet Names Server (http://earth-info.nga.mil), Wikimapia (http://www.wikimapia.org) and Google Earth (http://www.earth.google.com), which are freely available online. The identified location from one source was cross-checked against the other sources to confirm the consistency of the coordinates. Each collection area's coordinates were recorded using the Google Maps coordinate system. The recorded coordinates were then transferred to Microsoft Excel 2016 (Microsoft Corp., Redmond, WA, USA) to compile all the data for further analysis. All digital data and geographical coordinate were synchronized using the World Geodetic System (WGS1984; https://gisgeography.com/wgs84-world-geodetic-system/), which served as the* x*- (longitude or east–west) and* y*-coordinates (latitude or north–south), thereby allowing geographic positions to be expressed anywhere worldwide. The base map and all the environmental data were generated from freely available online sources reported in Table 1. The data were then exported and stored in ArcGIS 10.4.1 software (ESRI, Redlands, CA, USA) for further analysis.

**Table 1:** Remote sensing data and sources

Data type	Data source
Malaysia map (base map)	Department of Surveying and Mapping, Malaysia
Elevation	DIVA-GIS
Water bodies	Copernicus Global Land Service
Forest cover	Copernicus Global Land Service
Forest loss	Department of Geographical Science, University of Maryland
Temperature	WorldClim website

### Spatial cluster analysis

The distribution of the Leucosphyrus Group of *Anopheles* mosquitoes was mapped at the district and sub-district levels. Each coordinate was plotted as point features, creating a new geographic information system (GIS) layer representing the point locations of the mosquitoes. Distribution data on some Leucosphyrus Group of *Anopheles* mosquitoes from 1957 to 2021 were included in this map separately from the mosquito samplings conducted from 2019 to 2021 for the present study [8, 9, 11–14, 26, 27, 30–58].

The distribution of the Leucosphyrus Group of *Anopheles* mosquitoes was determined using the average nearest neighbor (ANN) analysis to calculate the ANN ratio (*R*) (Table 2) based on the observed average distance between the nearest mosquito location to determine the distribution pattern of the *Anopheles* mosquitoes.

**Table 2:** Average nearest neighbor ratio

Statistical formula	Explanation
$R = \frac{{\overline{D}_{\text{o}} }}{{\overline{D}_{\text{e}} }}$	$\overline{D}_{\text{o}}$ is the observed mean distance between each mosquito coordinated with the nearest neighboring mosquitoes $\overline{D}_{\text{e}}$ is the expected mean distance for the features determined as random pattern *R* refers to the radius used to measure the distances between features in a spatial dataset
$$\overline{D}_{\text{o}} = \frac{{\sum {\begin{array}{*{20}c} {{{n}}} \\ {{{i}}} \\ \end{array} } - 1{ }d_i }}{n}$$	*d*_*i*_ is the equal distance between each mosquito location and its nearest mosquitoes *i* is the nearest mosquito point *n* is the total number of mosquitoes *A* is the total study location SE is standardized expected nearest neighbor distance is the expected distance between features under the assumption of complete spatial randomness
$$\overline{D}_{\text{e}} = \frac{0.5}{{\sqrt {n/A} }}$$	
$$Z = \frac{{\overline{D}_{\text{o}} - \overline{D}_{\text{e}} }}{{{\text{SE}}}}$$	
$${\text{SE}} = \frac{{0.{26136}}}{{\sqrt {n^2 /A} }}$$	

The ANN analysis provides statistical values, such as *R*, *Z*-scores and *P* values. The distribution pattern of mosquitoes was used to determine the *R* value. When *R* < 1, the distribution of the *Anopheles* mosquitoes indicates clustering; when *R* > 1, the distribution pattern of the *Anopheles* mosquitoes is dispersing. The *Z*-scores were used to validate the calculated *R*-value to determine the significance of rejecting the null hypothesis.

### Inverse distance weighted interpolation method

An interpolation map was created to predict the risk area based on the Leucosphyrus Group of *Anopheles* mosquitoes’ coordinates by the inverse distance weighted (IDW) interpolation method. The IDW map was created from mosquito distribution data from 1957 up to 2021 and overlaid with the environmental variables, thus allowing the correlation of risk areas and the environmental factors to be observed. ArcGIS 10.4.1 (ESRI) software was used to perform all of the interpolation calculations. The IDW analysis formula was:

$$\frac{{\sum {\begin{array}{*{20}c} {{{n}}} \\ {{{i}}} \\ \end{array} } = 1 \frac{1}{d_i }v_i }}{{\sum {\begin{array}{*{20}c} {{{n}}} \\ {{{i}}} \\ \end{array} } { } = 1\frac{1}{d_i }}}$$

where, $\widehat{v}$ = estimated value; *V*_*i*_ = known value; and *d*_*i*_…, *d*_*n*_ = distances between the *n* data points and the estimated *n*.

### Statistical analysis

The logistic regression test was performed using SPSS software (Statistics 23; SPSS IBM Corp., Armonk, NY, USA) to identify the significant environmental variables linked to *Anopheles* mosquito abundance and develop a statistical risk model. The environmental variables were selected and entered into the regression model based on their significant values. The accuracy of the model was determined using regression analysis, which was divided into two groups: (i) sampling sites (218 locations); and (ii) random sampling sites (220 locations). The random sampling sites were created by ArcGIS 10.4.1 software (ESRI). Hosmer and Lemeshow goodness-of-fit tests were used to assess whether the model fits the observed data. The spatial autocorrelation test (Moran’s* I*-test) was applied to determine whether mosquito distribution patterns were clustered, scattered or randomly distributed. The * I*-test calculates the mean of each value at each site and compares it to the mean value of all locations. Moran’s* I*-test values range from − 1, which reflects a strong negative correlation, to + 1 which depicts a strong positive correlation. A Moran’s* I*-test value of 0 denotes a spatially random pattern.

Moran’s* I*-test was performed using ArcGIS 10.4.1 software (ESRI) [59]. The best fit logistic regression model was used to produce a predictive risk map based on the abundance of mosquitoes. The equation of the logistic regression model is denoted by:

$${\text{log}}\,{\text{odds}}\,{\text{of}}\,{\text{outcome}}\, = \,\beta 0\, + \,\beta 1x1\, + \,\beta 2x2\, + \,\beta 3x3\, + \, \cdots \, + \, \, \beta pxp\, = \,\beta^{\prime} x$$

where *β*_*i*_ is the regression coefficient for variable *x*_*i*_.

This equation can be re-written as:

$${\text{log}}\,{\text{odds}}\,{\text{of}}\,{\text{outcome}}\, = \,\log \left( {\frac{p}{1 - p}} \right)\, = \,{\text{logit}}(p)\, = \,\beta^{\prime} x$$.

(*p*) can be calculated by rearranging this equation as follows:

$$p = \frac{{\exp (\beta^{\prime} x)}}{{1 + \exp (\beta^{\prime} x)}}$$

## Results

### Distribution of Leucosphyrus Group of* Anopheles* mosquitoes from 1957 to 2021

**Fig. 2:**
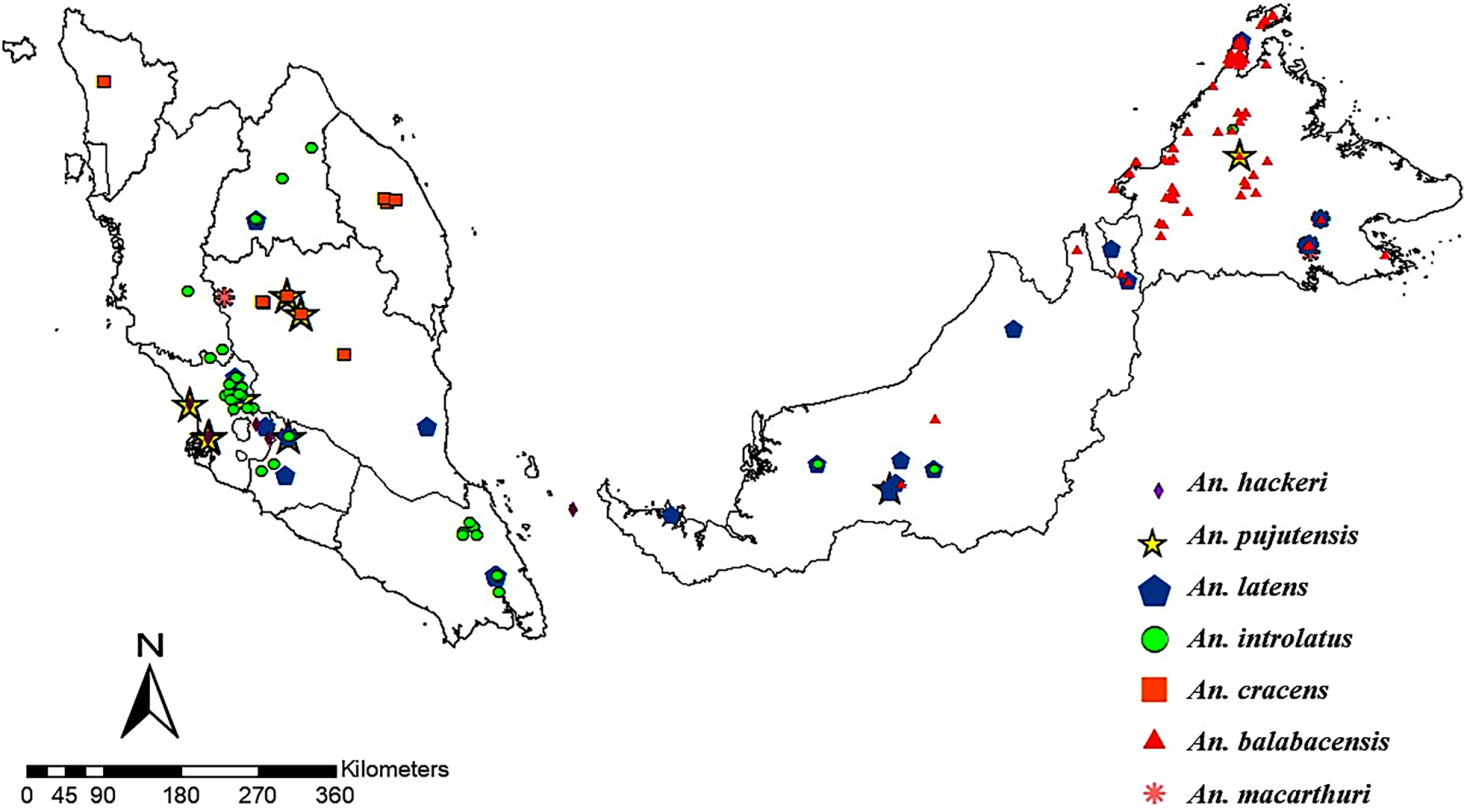
Overall distribution map of the Leucosphyrus Group of *Anopheles* mosquitoes from 1957 to 2021 in Malaysia

**Fig. 3:**
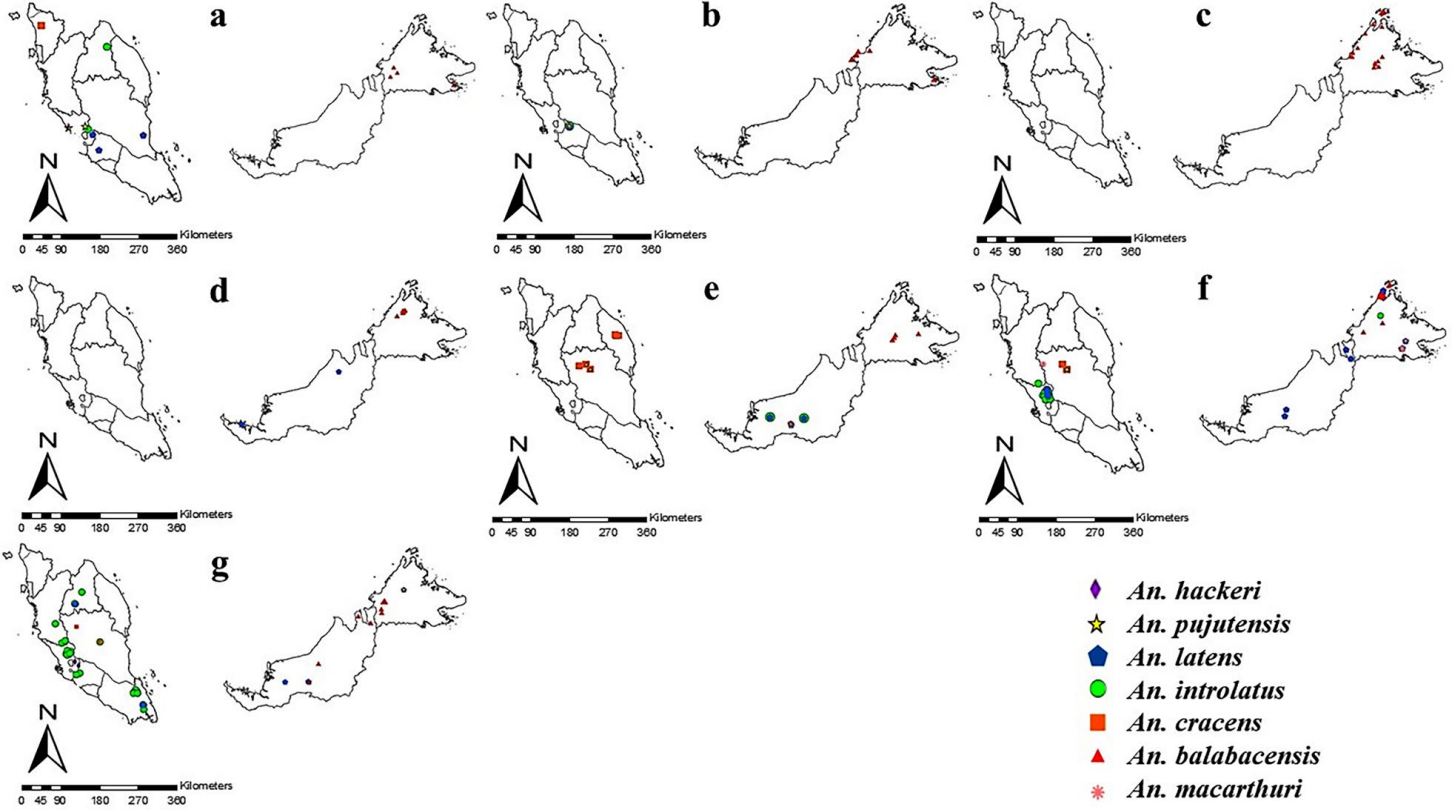
Distribution map of the Leucosphyrus Group of *Anopheles* mosquitoes from 1957 to 1967 (**a**), 1968–1977 (**b**), 1978–1987 (**c**), 1988–1997 (**d**), 1998–2007 (**e**), 2008–2017 (**f**) and 2018–2021 (**g**)

This database contains information on the whereabouts of the Leucosphyrus Group of *Anopheles* mosquitoes in Malaysia, and all locations have been successfully geopositioned (Additional file 1: Table S1). Figures 2 and 3 depict the overall geographical distribution of *Anopheles* mosquitoes and their distribution every 10 years. The ANN analysis reveals a consistent clustering pattern of *Anopheles* mosquitoes, with a nearest neighbor ratio (*R*) < 1 for overall distribution and every 10 years (Table 3). However, for the years 1988–1997, a dispersed pattern was observed (*R* > 1). The negative *Z*-score values indicate that clustering occurred randomly for almost every 10 years, except for the 1988–1997 period.

**Table 3:** Results from the average nearest neighbor analysis showing the distribution patterns of the *Anopheles* Leucosphyrus Group in Malaysia from 1957 to 2021

Year	*R*^a^	*P* value	*Z*-score	Pattern
1957–2021	0.20	0	− 22.50	Clustered
1957–1967	0.42	0	− 5.59	Clustered
1968–1977	0.37	< 0.01	− 4.33	Clustered
1978–1987	0.66	< 0.01	− 2.98	Clustered
1988–1997	2.80	0	9.71	Dispersed
1998–2007	0.07	0	− 6.93	Clustered
2008–2017	0.12	0	− 14.92	Clustered
2018–2021	0.24	0	− 10.13	Clustered

^a^*R* is the average nearest neighbor ratio. R < 1 indicates the distribution of the *Anopheles* mosquitoes is clustered; *R* > 1 indicates the distribution pattern of the *Anopheles* mosquitoes is dispersed

### Spatial interpolation

**Fig. 4:**
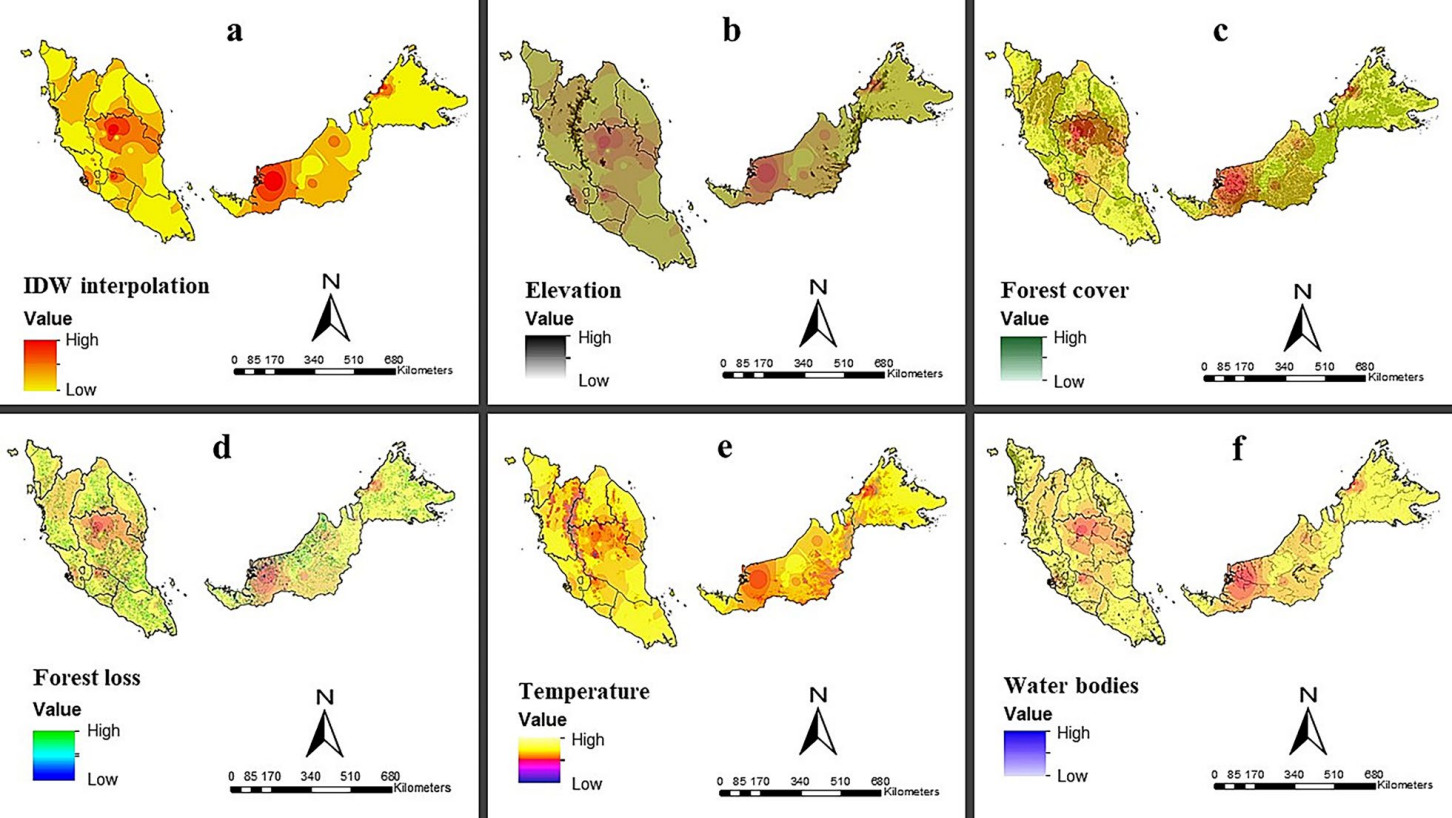
Interpolated distribution of vectors illustrating zonation of high and low areas (**a**), vector distribution overlaid with elevation (**b**), forest cover (**c**), forest loss (**d**), temperature (**e**) and water bodies (**f**). IDW, inverse distance weighted (interpolated method)

The IDW interpolation method was utilized to analyze spatial interpolation based on vector data collected between 1957 and 2021. Predictions for *Anopheles* mosquito abundance were categorized by color, ranging from low (yellow) to moderate (orange) to high (red). To enhance the visual representation, maps displaying environmental factors, such as forest loss, forest cover, elevation, temperature and water bodies, were overlaid onto the prediction map. The IDW results revealed that a high number of vectors could be observed in the southwest part of Sarawak (Betong, Kapit, Bintulu, Miri, Sibu, Mukah, Sri Aman, Song and Sarikei), Kedah (Baling), Perak (Hulu Perak and Kerian), Pahang (Kuala Lipis, Jerantut, Bentong, Temerloh, Bera, Maran and Kuantan), Negeri Sembilan (Jelebu, Tampin and Jempul), Kelantan (Gua Musang, Kota Baharu, Jeli, Pasir Mas and Tumpat), Terengganu (Hulu Terengganu, Dungun and Kemaman), Selangor (Kuala Langat, Kuala Selangor and Hulu Selangor), Johor (Mersing), Melaka (Alor Gajah and Jasin) and the northwest part of Sabah (Kudat, Ranau, Penampang, Papar, Tuaran and Beaufort). In contrast, most parts of Johor, Sabah, Perlis, Penang, Kelantan, and Terengganu had low vector abundance (Fig. 4).

High elevations in Pahang (Titiwangsa range), Sarawak (Sarawak-Kalimantan border range and northeast part) and Sabah (northwest and southwest part) were locations with moderate and low mosquito abundance (Fig. 4a). The orange and red zones of Sabah, Sarawak, Pahang, Perak, Kelantan, Terengganu and Kedah are associated with high forest cover (Fig. 4c). Areas with high and moderate mosquito abundance exhibit noticeable tree loss across Malaysia (Fig. 4d). Low temperature (Fig. 4e) was linked with high elevations in Malaysia (Fig. 4b). Water bodies were also present near zones with moderate and high mosquito abundance (Fig. 4f). Maps of the environmental factors are shown in Additional file 2: Figure S1.

### Statistical and spatial analysis

Based on ecological factors, this study used logistic regression analysis to predict the abundance of *Anopheles* mosquitoes in unsampled areas. The results showed that elevation, forest cover, forest loss and temperature were significant factors affecting the distribution of the Leucosphyrus Group of *Anopheles* mosquitoes (Table 4). However, water bodies were excluded from the final model as they were not statistically significant. The model's accuracy in predicting sampled and non-sampled areas was 75.3%, with 73% accuracy for sampled areas and 77.6% accuracy for non-sampled areas. The model's accuracy was reliable, as most non-sampled areas were predicted with *P* < 0.5, and sampled areas were predicted at *P* > 0.5. The Hosmer and Lemeshow goodness-of-fit value was statistically significant, indicating that the model's estimates fit well with the data. The spatial distribution of mosquitoes was evaluated using Moran’s* I*-index, which showed a significantly positive spatial autocorrelation for the distribution of the Leucosphyrus Group of *Anopheles* within districts, indicating that mosquito distribution was more spatially clustered.

**Table 4:** Regression coefficients used to estimate the distribution of *Anopheles* mosquitoes

Characteristic	Coefficient estimate (β)	Standard error of estimate	Odds ratio (95% CI)	*P* value^a^
Constant	− 28.472	5.070	–	–
Forest cover	0.022	0.003	1.02 (1.02–1.03)	0.000
Temperature	− 0.302	0.110	0.74 (0.60–0.92)	0.006
Elevation	0.006	0.001	1.01 (1.00–1.01)	0.000
Forest loss	0.068	0.023	1.07 (1.024–1.12)	0.003
Water bodies	− 0.054	0.032	0.95 (0.89–1.01)	0.090

*CI* Confidence Interval

^a^Significance level was set at *P* < 0.05

Hence, our logistic regression model is:

$$\begin{aligned} {\text{Log}}\,{\text{odds}}\,{\text{of}}\,{\text{outcome}} & = \,\log \left( {\frac{P}{1 - P}} \right) \\ & = \, - {28}.{472}\, + \,0.0{22}*{\text{f}}.{\text{cover}}\, + \, - \,0.{3}0{2}*{\text{temp}}\, + \,0.00{6}*{\text{elev}}_+ 0.0{68}*{\text{f}}.{\text{loss}} \\ &= \beta^{\prime} x \\ \end{aligned}$$

Which can be arranged as follows:

$$P = \frac{{{\text{exp}}( - \,{28}.{472}\, + \,0.0{22}*{\text{f}}.{\text{cover}}\, + \, - \,0.{3}0{2}*{\text{temp}}\, + \,0.00{6}*{\text{elev}}_+ 0.0{68}*{\text{f}}.{\text{loss}}}}{{{1}\, + \,{\text{exp}}( - \,{28}.{472}\, + \,0.0{22}*{\text{f}}.{\text{cover}}\, + \, - \,0.{3}0{2}*{\text{temp}}\, + \,0.00{6}*{\text{elev}}_+ 0.0{68}*{\text{f}}.{\text{loss}}}}$$

### Modeled distribution of Leucosphyrus Group of *Anopheles*

**Fig. 5:**
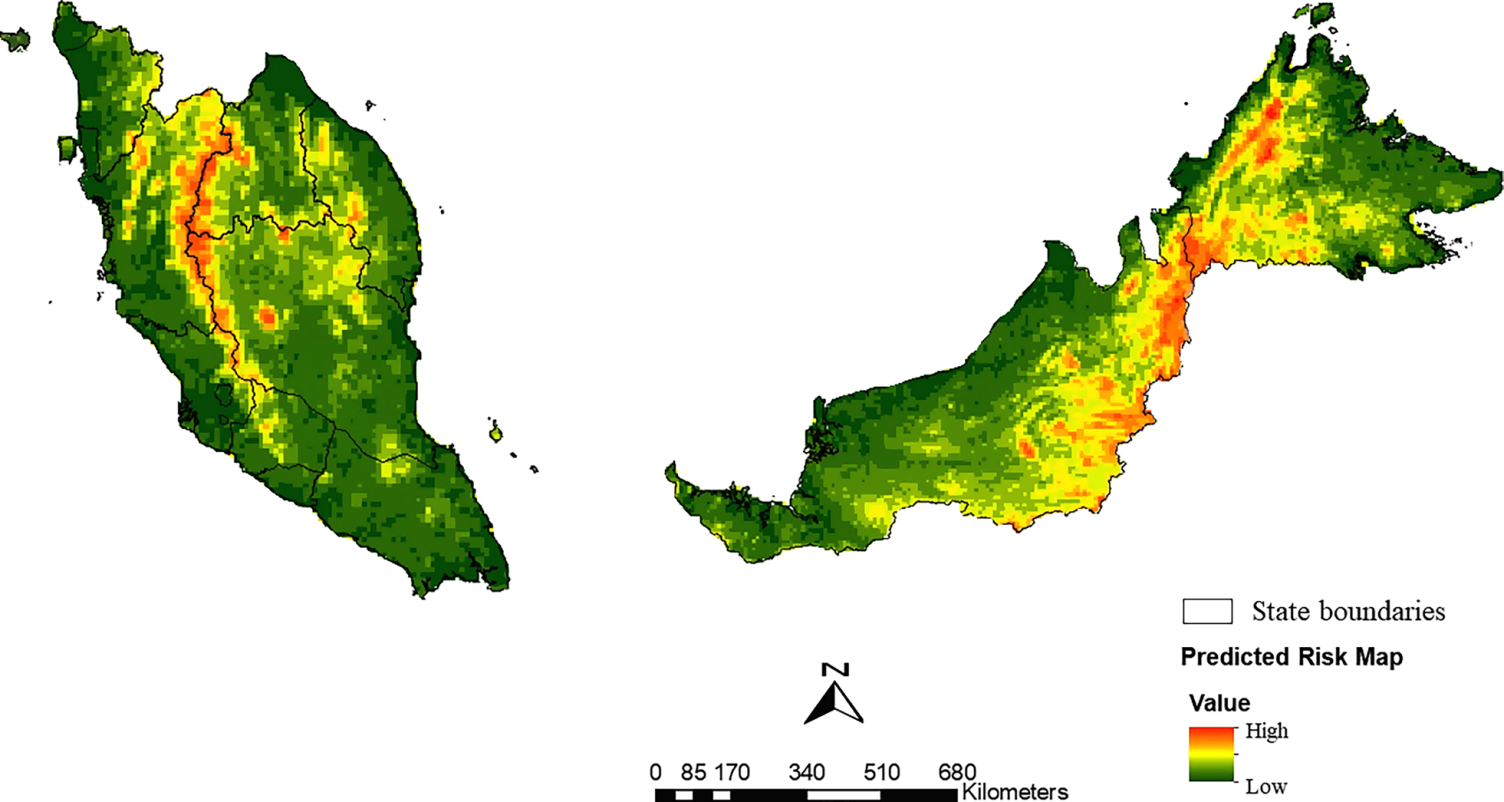
Predicted risk map of Leucopshyrus Group of *Anopheles* mosquitoes in Malaysia as derived from the logistic regression model

A logistic regression model was used to predict the distribution of the Leucosphyrus Group of *Anopheles* mosquitoes based on environmental factors such as elevation, forest cover, forest loss and temperature. The results showed a higher predicted distribution of *Anopheles* mosquitoes (indicated by red–orange coloration) in certain areas, including the Titiwangsa range, central and northern parts of Peninsular Malaysia, Kudat Division, West Coast Division, Interior Division and Tawau Division of Sabah, as well as Kapit, Lawas, Marudi, Belaga, Song and Sri Aman of Sarawak. Meanwhile, the predicted distribution of *Anopheles* mosquitoes was lower (indicated by shades of green coloration) in the southern part of Peninsular Malaysia (mainly in Johor, Perlis, Melaka, Selangor, Kuala Lumpur and Penang), the eastern part of Sabah (mainly in Pitas, Sandakan, Sempurna, Beluran and Kuala Penyu), and the western region of Sarawak (primarily Kuching, Serian, Betong and Mukah) (Fig. 5).

## Discussion

Using GIS, in this study we have outlined the distribution of the *Anopheles* Leucosphyrus Group of mosquitoes in Malaysia. The information gathered from previous published studies and from current field data is crucial for identifying and managing malaria vectors. The study also suggests that this approach could facilitate the planning and advancement of vector management strategies. The results show significant geographic variation in the distribution of *Anopheles* throughout Malaysia, which opens the door to an opportunity to effectively target prevention efforts where they are needed most, particularly when resources are limited.

We found that the distribution of malaria vectors, particularly in Malaysian Borneo, has remained largely unchanged from 1957 to 2021. The primary simian malaria vectors vary by region, with *An. balabacensis* prevalent in Sabah and Sarawak [11, 12, 60], *An. latens* in Sarawak [10, 53], *An. cracens* in Pahang [8, 9] and *An. introlatus* occurring in Peninsular Malaysia and also observed in Sarawak [12]. However, *An. introlatus* was only confirmed as a vector in Selangor [13]. Our distribution maps illustrate these findings. Recent studies have also identified *An. introlatus* as a vector of simian malaria in Peninsular Malaysia [61].

Analysis of the geospatial data from 1957 to 2021 revealed that *Anopheles* mosquitoes tended to cluster in certain areas. This was shown in the ANN analysis, which also revealed clustering patterns among different *Anopheles* spp. of the Leucosphyrus Group. There were clusters of > 2 species in Selangor from 1957 to 1967, in Negeri Sembilan from 1968 to 1977) and in Sarawak from 1998 to 2007. *Anopheles hackeri, Anopheles pujutensis, An. introlatus*, and *An. latens* were found in Selangor, while *An. pujutensis*, *Anopheles macarthuri, An. introlatus* and *An. latens* were found in Negeri Sembilan and Sarawak. These species thrived in these areas due to the presence of their ideal habitat conditions, such as optimal temperature for larval and adult mosquito development [62], clean water bodies for breeding sites [63] and proximity to forested or deforested areas for easy access to humans and animals for blood meals [64, 65].

Developing an interpolation map based on species distribution datasets can help identify vector-prone areas and plan effective vector control programs. Some entomological and vector studies have used interpolation techniques, such as the IDW method, to predict mosquito species abundance in non-sampled areas [66–70]. Despite limited data in some parts of the country, the interpolation map estimates vector abundance. In the present study, high vector abundances were observed in Sarawak's southwestern region, Pahang’s northern region and Sabah's northwestern region. Environmental variables, such as forest cover, deforestation, elevation, water bodies and temperature, can significantly influence vector abundance [62–65, 71, 72].

The *Anopheles* Leucosphyrus Group of mosquitoes are forest-dwelling, typically found in forested and agricultural settings [13]. Furthermore, *Anopheles* larval habitats are found in highly shaded, clean, natural water pockets or puddles near rivers [32]. Thus, the predicted high vector abundance is near forested and water bodies areas. Since *Anopheles* lives in a shaded, humid and moist environment [73], this environment offers clean and suitable water bodies for *Anopheles* mosquito breeding sites. With deforestation, long-tailed macaques (*Macaca fascicularis*) and pig-tailed macaques (*Macaca nemestrina*) [74], the natural host of simian malaria in Malaysia, have migrated to the forest fringes, and these mosquitoes may have trailed them there and subsequently colonized forest fringes. Therefore, vector abundance is high where deforestation is noted since the vector is localized in the disturbed natural environment where it is observed to have high biting rates in agricultural areas and forest fringes [9, 53, 75]. It is possible that the Leucosphyrus Group of mosquitoes were not prevalent during human malaria entomological studies [26, 76] conducted in Peninsular Malaysia because they may have been residing in the densely forested regions during that time.

The presence of vectors in a particular habitat is affected by elevation and temperature. Elevated areas typically have lower temperatures, which can limit the reproduction and growth of *Anopheles* mosquitoes, resulting in fewer of them being observed [77, 78]. As a result, this study found that vector abundance was lower in areas with both high elevation and low temperatures. The high number of vectors noted in the IDW interpolation map was thus influenced by factors such as forest loss, forest cover, elevation, and water bodies and temperature.

The logistic regression model for predicting the *knowlesi* malaria vector performed well when analyzing the covariate data. The resulting predictive map also aligns well with the actual *knowlesi* malaria cases recorded in a previous study [79]. The risk of contracting *knowlesi* malaria is higher in forested and deforested areas, with the highest vector population. This was determined through an interpolation map that overlaid forest cover and forest loss covariates. Additionally, the predicted high-risk zones for *knowlesi* malaria are mainly around the Titiwangsa range and central-northern region of Peninsular Malaysia [79]; the same pattern was noticed from the predictive vector map from this study. The association between the predictive vector and human case maps would further support the model's accuracy and reliability.

Furthermore, most of these published data were published more than a few years back and applying that to the given day is doubtful. Despite these drawbacks, the existing data provide a reasonably estimated distribution of simian malaria vectors in Malaysia. Although the predictive model can be used to visualize the entire distribution of vectors based on environmental parameters, some variables also give valuable information for the prediction, such as the distribution of simian malaria infection and case characteristics like gender, age, and occupation. Indeed, such details were not included in the present mapping survey, but they could be a focus for future research. Consequently, we must know that this data is difficult to obtain on a broad spatial scale. Apart from such limitations, the information in the current database can assist in identifying and emphasizing where such information is lacking and, therefore, may collect the necessary data for further research on the geographical distribution of vectors in the country. In addition, the study used freely available databases to create a predictive map, a cost-effective way to support entomological surveillance efforts.

This map can help identify areas or populations with the greatest needs. It considers factors such as forest cover and loss, which can affect the abundance of disease-carrying insects. The map predicts that Kedah, Pahang, Kelantan, Terengganu, Selangor, Negeri Sembilan, Sabah, and Sarawak likely have the highest vector distribution. These areas have increased forest cover and forest loss, more water bodies, low elevation, and moderate temperatures.

Forest cover effects on anopheline abundances during the dry and wet seasons can be linked to the behavior of adults and larvae formations [80]. Water quality is influenced by forest cover through shading, organic matter inputs, and erosion processes [81]. These factors impact water quality and facilitate vector breeding sites [82]. Vectors and their hosts correlate with the forest. Some mosquitos are zoophilic and feed on animals [83], commonly more abundant in forested areas. When forested areas are replaced by agricultural land, the plants can still offer the bushy cover that some *Anopheles* mosquito species or larval development stages require. Thus, it increases the rate of mosquito densities [84–86]. *Anopheles balabacensis*, the primary simian malaria vector in Sabah [11], is a forest-dwelling species with larval development that prefers humid, shaded water conditions [87]. *Anopheles balabacensis* abundances in Sabah have recently been higher in the disturbed, cleared forest, plantations, and farms than in undisturbed primary and secondary forested areas. An example of how land use can affect the ecology of a vector can be seen in Sabah [11, 36, 39].

The man biting rate of *An. donaldi* and *An. letifer* was higher in forested areas of Sarawak than in villages [37]. However, when deforestation occurred and palm oil plantations were established over four years, the vector population declined [88]. On the other hand, another study in Peninsular Malaysia found that the man-biting rate of the *Anopheles* Leucospyrus Group of mosquitoes was higher in forested areas and remained relatively similar in agricultural areas in Sungai Dara (Perak), Kem Sri Gading, (Pahang), Kampung Lalang (Kelantan) and Bukit Tinggi and Gunung Panti (Johor)[61]. These findings suggest that forest clearing disrupts habitats and brings different ecosystems closer together, creating new environments at the forest edge [89]. Deforestation can also alter the microclimate, vegetation, and soil composition, bringing a new environment for vectors [90, 91] and adapting the new ecology. Therefore, the abundance of vectors tends to be higher in areas where there has been forest loss.

The immature stages of malaria vector mosquitoes, such as eggs, larvae, and pupae, can be found in surface water [92]. Using satellite data to monitor these water bodies is valuable in identifying the source of these disease-carrying mosquitoes. However, it's important to note that the assumption that vector abundance is negatively correlated with water bodies may not be accurate. This is because most of the sampled locations were near water bodies but not within them, which failed to obtain the necessary data for analysis. To improve future studies, conducting *Anopheles* larval surveys and including them in predictive maps is crucial, as larval habitat presence is a direct approach to predicting vector density. It is essential for researchers to carefully evaluate the study's outcomes, as one study found similar limitations and discussed ways to improve their analysis [92]. This limitation highlights the importance of identifying *Anopheles* larval sites near sampling areas, considering the prevalence of water body characteristics, as female *Anopheles* mosquitoes require water to complete their life cycle [93].

The density of vectors is generally lower in the highlands compared to the nearby lowlands, as observed in this study [94–96]. The elevation and temperature are linked, and as the temperature decreases with increasing elevation, malaria vectors' density and species diversity may also vary [96]. Temperature also affects the development rates of juveniles, the duration of the gonotrophic cycle, and the survival of both juvenile and adult phases at an optimal temperature [71]. *Anopheles* mosquitoes’ survival is directly influenced by environmental temperature during juvenile and adult stages. A warmer atmosphere promotes rapid growth and smaller adults [98]. High temperatures speed up the evaporation rate of water pools and reduce pool lifetime, thus, mosquito immatures have limited time to reach the adult phase. Studies have shown that *Anopheles* larvae cannot survive at temperatures higher than 35 °C [62, 97]. Higher temperatures can speed up blood meal digestion, shorten gonotrophic cycles, and alter mosquitoes' reproduction ability [98]. At temperatures below 17 °C, malaria vectors fail to survive [99]. Therefore, vector abundance is low at both high and low temperatures as they require an ideal temperature for their life history.

## Conclusion

This study provides important information about the distribution of simian malaria vectors in Malaysia from 1957 to 2021. The study also includes a predictive map of vector abundance based on environmental factors. This information can help reduce malaria by allowing authorities to focus on areas with high transmission rates. Understanding the environment is crucial because it can increase or decrease the number of vector breeding sites. According to the study, water bodies, areas with ideal temperatures, low-lying areas, deep forests, and deforested zones have high vector density. The study used freely available databases to create a predictive map, a cost-effective way to support entomological surveillance efforts. Health professionals in areas with "very high" and "high" vector abundance should take targeted measures to reduce vector populations and human cases. In doing so, they can maximize the benefits of their efforts and optimize the impact of vector control interventions.

### Supplementary Information

**Additional file 1: Table S1.** Vectors throughout Malaysia and the sites recorded from 1957 to 2022.

**Additional file 2: Figure S1.** Maps showing the elevation, forest cover, forest loss, temperature and water bodies of Malaysia.

## Data Availability

The datasets generated from the journals during the current study are available in online.

## Abbreviations

ANN: Average nearest neighbor
GIS: Geographic information system
IDW: Inverse distance weighted
RS: Remote sensing
