# Supplementary material for: Spatial analyses of Plasmodium knowlesi vectors with reference to control interventions in Malaysia
Source: Parasit Vectors. 2023 Oct 9;16:355. doi: 10.1186/s13071-023-05984-x (PMC10563288; doi:10.1186/s13071-023-05984-x)
Supplement: Supplementary file 1 — Additional file 1: Table S1. Vectors throughout Malaysia and the sites recorded from 1957 to 2022. [file 13071_2023_5984_MOESM1_ESM.docx]

**Additional file 1.**

**Table S1: The vectors throughout Malaysia and the places from 1957 to 2022.**

| Species | Sites | State, Districts | Year | Coordinates |
| --- | --- | --- | --- | --- |
| *An. introlatus* | Ulu Gombak | Selangor, Gombak | 1964 | 3°18'00.0"N 101°46'52.1"E |
|  | Bukit Kertau | Pahang, Chenor | 1970 | 3°04'05.0"N 102°05'59.1"E |
|  | Ulu Kelantan | Kelantan, Kuala Krai | 1961 | 5°31'39.2"N 102°17'08.3"E |
|  | Ulu Sungai Yong | Sarawak, Kapit | 2005-2006 | 2°12'10.5"N 112°01'55.3"E |
|  | Lubok Loh Yong | Sarawak, Kapit | 2005-2006 | 2°09'16.1"N 113°20'39.0"E |
|  | Kuala Kubu Bharu | Selangor, Hulu Selangor | 2009-2013 | 3°34'01.2"N 101°38'52.1"E |
|  | Ladang Getah Rasa | Selangor, Hulu Selangor | 2012 | 3°29'58.7"N 101°35'09.7"E |
|  | Ulu Kalong | Selangor, Btg Kali | 2013 | 3°24'31.8"N 101°32'41.6"E |
|  | Ladang Getah Widuri | Selangor, Bkt Beruntung | 2013 | 3°25'56.0"N 101°34'54.6"E |
|  | Menara Celcom Gesir | Selangor, Hulu Bernam | 2013 | 3°43'40.2"N 101°25'03.6"E |
|  | Serendah Golf | Selangor, Serendah | 2013 | 3°43'40.2"N 101°25'03.6"E |
|  | Kg. Baru Ulu Rening | Selangor, Btg Kali | 2013 | 3°28'59.0"N 101°41'14.9"E |
|  | Kg. Ulu Kalong, Ulu Yam Baru | Selangor, Btg Kali | 2013 | 3°25'32.5"N 101°40'12.4"E |
|  | Serendah Golf | Selangor, Serendah | 2013 | 3°22'18.6"N 101°35'20.8"E |
|  | Village | Sabah, Ranau | 2017 | 05º58’N,116º42E |
|  | Commonwealth Recreational Park | Selangor, Rawang | 2011-2012 | 3°17'44.4"N 101°36'49.6"E |
|  | Sedayu Recreational Park | Selangor, Gombak | 2011-2012 | 3°18'27.5"N 101°44'08.2"E |
|  | Sg. Sendat Recreational Park | Selangor, Hulu Yan | 2011-2012 | 3°24'15.1"N 101°41'05.3"E |
|  | Gunung Panti | Johor, Kota Tinggi | 2019 | 1°52'19.0"N 103°52'23.7"E |
|  | Gunung Panti | Johor, Kota Tinggi | 2019 | 1°52'01.6"N 103°52'40.3"E |
|  | Gunung Panti | Johor, Kota Tinggi | 2020 | 1°52'18.4"N 103°52'23.2"E |
|  | Kg. Sri Delima | Johor, Kota Tinggi | 2019 | 1°52'17.7"N 103°52'25.4"E |
|  | Hutan Lenggor | Johor, Mersing | 2019-2020 | 2°19'41.0"N 103°38'41.0"E |
|  | Hutan Lenggor | Johor, Mersing | 2019-2020 | 2°17'43.0"N 103°40'01.0"E |
|  | Hutan Lenggor | Johor, Mersing | 2019-2020 | 2°17'45.0"N 103°39'35.0"E |
|  | Hutan Lenggor | Johor, Mersing | 2019-2020 | 2°17'14.0"N 103°40'20.0"E |
|  | Hutan Lenggor | Johor, Mersing | 2019-2020 | 2°19'41.0"N 103°38'41.0"E |
|  | Hutan Lenggor | Johor, Mersing | 2019-2020 | 2°13'28.3"N 103°42'10.8"E |
|  | Hutan Lenggor | Johor, Mersing | 2019-2020 | 2°13'29.1"N 103°42'10.9"E |
|  | Kem Microwave | Johor, Mersing | 2019-2020 | 2°13'29.3"N 103°42'10.3"E |
|  | Kem Microwave | Johor, Mersing | 2019-2020 | 2°17'44.4"N 103°39'35.8"E |
|  | Kem Microwave | Johor, Mersing | 2019-2020 | 2°13'30.0"N 103°42'10.6"E |
|  | Kem Microwave | Johor, Mersing | 2019-2020 | 2°17'47.9"N 103°39'52.5"E |
|  | Kem Microwave | Johor, Mersing | 2019-2020 | 2°17'46.8"N 103°39'58.4"E |
|  | Kongsi Balak | Johor, Mersing | 2019-2020 | 2°13'28.3"N 103°42'10.8"E |
|  | Kg. OA Berasau | Johor, Kluang | NA | 2°13'01.3"N 103°35'13.0"E |
|  | Kg. OA Punjut | Johor, Kluang | 2020 | 2°14'19.5"N 103°34'46.6"E |
|  | Kg. Lalang | Kelantan, Gua Musang | 2019-2020 | 4°54'32.6"N 101°48'58.6"E |
|  | Kg. Lebur Jaya | Kelantan, Gua Musang | 2019 | 4°55'14.0"N 101°48'47.9"E |
|  | Kg. Dusun Durian | Kelantan, Gua Musang | 2020 | 5°15'47.8"N 102°01'46.5"E |
|  | Kem Sri Gading (UiTM) | Pahang, Jengka | 2020-2021 | 3°45'39.8"N 102°34'17.4"E |
|  | Kg. Sg Dara (Loji) | Perak, Mualim | 2020-2022 | 3°47'50.6"N 101°31'44.7"E |
|  | Kem Draco | Perak, Mualim | 2022 | 4°18'26.9"N 101°13'39.9"E |
|  | Hutan Lenggeng | Negeri Sembilan, Seremban | 2021 | 2°49'45.1"N 101°58'05.5"E |
|  | Kebun Durian Tekir(near Kg. OA asli tekir) | Negeri Sembilan, Seremban | 2021 | 2°46'21.7"N 101°51'33.1"E |
| *An. latens* | Ulu Lui | Selangor, Hulu Langat | 1964 | 3°09'06.0"N 101°54'19.2"E |
|  | Ulu Bendol | Negeri Sembilan, Seremban | 1964 | 2°43'50.2"N 102°04'19.3"E |
|  | Jabor Valley | Pahang | 1965 | 3°08'38.8"N 103°16'59.6"E |
|  | Bukit Kertau | Pahang, Chenor | 1970 | 3°04'05.0"N 102°05'59.1"E |
|  | Tinjar | Sarawak, Baram | 1991 | 3°43'29.6"N 114°14'09.6"E |
|  | Kuching Metropolitan Area | Sarawak, Kuching | 1997 | 1°38'14.9"N 110°23'29.0"E |
|  | Lubok Loh Yong | Sarawak, Kapit | 2005-2006 | 2°09'16.1"N 113°20'39.0"E |
|  | Ulu Sungai Yong | Sarawak, Kapit | 2005-2006 | 2°12'10.5"N 112°01'55.3"E |
|  | Forest | Sarawak, Kapit | 2005-2006 | 1°56'43.4"N 112°50'09.7"E |
|  | Sg. Sut | Sarawak, Kapit | 2006 | 1°54'00.0"N 112°50'52.1"E |
|  | Forest | Sarawak, Kapit | 2008 | 2°00'00.0"N 112°54'52.1"E |
|  | Ulu Sg Yong | Sarawak, Kapit | 2008 | 2°15'00.0"N 112°57'52.1"E |
|  | Kuala Kubu Bharu | Selangor, Hulu Selangor | 2009-2013 | 3°34'01.2"N 101°38'52.1"E |
|  | Kg. Ulu Kalong, Ulu Yam Baru | Selangor, Btg Kali | 2012 | 3°25'32.5"N 101°40'12.4"E |
|  | Tajau Laut | Sabah, Kudat | 2013- 2015 | 6°57′44.5″N, 116°48′56.3″E |
|  | Danum Valley Field Centre | Sabau, Tawau | 2014 | 4°58′N, 117°42′E |
|  | Benta Wawasan oil palm plantation | Sabau, Tawau | 2014 | 4°40′N, 117°32′E |
|  | Benta Wawasan oil palm plantation | Sabau, Tawau | 2014 | 4°41′N, 117°34′E |
|  | Long Luping | Sabau, Tawau | 2015 | 4° 16′ 18′′ N, 115° 31′ 49′′ E |
|  | Long Tengoa village | Sabau, Tawau | 2015 | 4° 37′ 5′′ N, 115° 20′ 23′′ E |
|  | Paus | Sabah, Ranau | 2015-2018 | 5°41′N, 116°47′E |
|  | Gunung Panti | Johor, Kota Tinggi | 2019 | 1°52'19.0"N 103°52'23.7"E |
|  | Gunung Panti | Johor, Kota Tinggi | 2019 | 1°52'14.8"N 103°52'12.2"E |
|  | Gunung Panti | Johor, Kota Tinggi | 2019 | 1°52'01.6"N 103°52'40.3"E |
|  | Gunung Panti | Johor, Kota Tinggi | 2019 | 1°52'19.4"N 103°52'23.4"E |
|  | Kg. Lalang | Kelantan, Gua Musang | 2019-2020 | 4°54'32.6"N 101°48'58.6"E |
|  | Rumah Sewa Panto | Sarawak, Kapit | 2020 | 2°01'03.0"N 112°56'51.5"E |
|  | Kem Kayu Balak Merarap | Sarawak, Lawas | 2020 | 4°21'09.9"N 115°27'36.5"E |
|  | Simpang Utong | Sarawak, Kapit | 2020 | 2°00'04.0"N 112°58'21.2"E |
|  | Kg. Sawang | Sarawak, Kapit | 2020 | 2°00'30.6"N 112°00'26.3"E |
|  | Taman Ixora | Sarawak, Kapit | 2020 | 2°00'54.2"N 112°56'15.6"E |
| *An. cracens* | 20th Mile Padang Besar Rd | Perlis | 1965 | 6°06'07.0"N 100°30'59.1"E |
|  | Kg. Basoh | Terengganu, Kuala Berang | 2003 | 5°05'00.0"N 103°00'52.1"E |
|  | Sg. Ular | Pahang, Kuala Lipis | 2007 | 4°15'06.0"N 102°04'09.0"E |
|  | Kg. Dura | Terengganu, Kuala Berang | 2007 | 5°04'00.0"N 102°56'00.0"E |
|  | Kg. Tapah | Terengganu, Kuala Berang | 2007 | 5°06'00.0"N 102°54'52.1"E |
|  | Serunai Mela | Pahang, Kuala Lipis | 2007-2008 | 4° 7.0'N, 102° 11.9'E |
|  | Sg. Ular Village | Pahang, Kuala Lipis | 2007-2008 | 4°12.673’N, 101°53.127’ E |
|  | Sg. Ular | Pahang, Kuala Lipis | 2007-2008 | 4°15.7'N, 102°4.8'E |
|  | Serunai Mela | Pahang, Kuala Lipis | 2017 | 4° 7.0'N, 102° 11.9'E |
|  | Serunai Mela | Pahang, Kuala Lipis | 2017 | 4° 15.7'N, 102°4.8'E |
|  | Kg. Dusun Durian | Pahang, Kuala Lipis | 2015 | 4°12'53.0"N 101°51'50.0"E |
|  | Kem Sri Gading (UiTM) | Pahang, Jengka | 2020-2021 | 3°45'39.8"N 102°34'17.4"E |
| *An. balabacensis* | Lingan | Sabah, Banggi Island | 1986 | 116° 2' E 5° 46' N |
|  | Kapitangan | Sabah, Banggi Island | 1986 | 7°15'00.0"N 117°09'52.1"E |
|  | North Keningau | Sabah, Keningau | 1957 | 5°24'57.3"N 116°20'46.4"E |
|  | North Keningau | Sabah, Keningau | 1959 | 5°24'57.3"N 116°20'46.4"E |
|  | North Keningau | Sabah, Keningau | 1960 | 5°24'57.3"N 116°20'46.4"E |
|  | North Keningau | Sabah, Keningau | 1962 | 5°24'57.3"N 116°20'46.4"E |
|  | North Keningau | Sabah, Keningau | 1963 | 5°24'57.3"N 116°20'46.4"E |
|  | North Keningau | Sabah, Keningau | 1967 | 5°24'57.3"N 116°20'46.4"E |
|  | North Keningau | Sabah, Keningau | 1968 | 5°24'57.3"N 116°20'46.4"E |
|  | South Keningau | Sabah, Keningau | 1957 | 5°03'05.3"N 116°11'37.6"E |
|  | Tenom | Sabah, Tenom | 1957 | 4°54'07.2"N 115°55'13.9"E |
|  | Tenom | Sabah, Tenom | 1958 | 4°54'07.2"N 115°55'13.9"E |
|  | Semporna | Sabah, Semporna | 1965 | 4°33'47.3"N 118°25'41.5"E |
|  | Semporna | Sabah, Semporna | 1976 | 4°33'47.3"N 118°25'41.5"E |
|  | Kuala Penyu | Sabah, Kuala Penyu | 1970 | 5°28'25.6"N 115°32'35.6"E |
|  | Semporna | Sabah, Semporna | 1970 | 5°38'56.4"N 116°02'13.7"E |
|  | Kiaru | Sabah, Kuala Penyu | 1976-1977 | 5°36'20.9"N 115°36'54.2"E |
|  | Menunggang | Sabah, Kuala Penyu | 1976-1977 | 5°29'50.7"N 115°32'16.6"E |
|  | Menumbok Darat | Sabah, Kuala Penyu | 1976-1977 | 5°18'13.5"N 115°21'50.6"E |
|  | Lingan | Sabah, Papar | 1978 | 5°37'24.2"N 115°59'59.7"E |
|  | Lingan | Sabah, Papar | 1986 | 5°46'00.0"N 116°02'00.0"E |
|  | Kapitangan | Sabah, Bangi Island | 1986 | 7°15'00.0"N 117°10'00.0"E |
|  | Telupid | Sabah, Telupid | 1987 | 5°37'31.2"N 117°05'47.3"E |
|  | Kudat | Sabah, Kudat | 1987 | 6°48'24.1"N 116°45'41.5"E |
|  |  | Sabah, Kota Kinabalu | 1985 | 5°57'02.8"N 116°12'07.9"E |
|  | Kapitangan | Sabah, Bangi Island | 1984 | 7°15'00.0"N 117°10'00.0"E |
|  | Forest | Sabah, Papar | 1986 | 5°38'00.0"N 115°56'00.0"E |
|  | Limbuak Darat | Sabah, Bangi Island | 1982-1983 | 7°11'52.0"N 117°05'15.0"E |
|  | Limbuak Laut | Sabah, Bangi Island | 1982-1983 | 7°12'33.1"N 117°03'52.6"E |
|  | Limbuak Cattle Farm | Sabah, Bangi Island | 1982-1983 | 6°28'03.1"N 116°28'47.5"E |
|  | Penagah | Sabah, Kinabatangan | 1982-1983 | 5°16'11.4"N 116°58'25.9"E |
|  | Mengkawago | Sabah, Kinabatangan | 1982-1983 | 5°14'27.1"N 116°47'52.1"E |
|  | Minusoh | Sabah, Kinabatangan | 1982-1983 | 5°20'52.5"N 116°50'53.4"E |
|  | Langkabong | Sabah, Kinabatangan | 1982-1983 | 5°27'37.0"N 116°56'48.4"E |
|  | Menamam | Sabah, Kinabatangan | 1982-1983 | 5°23'55.7"N 116°50'25.2"E |
|  | Kg. Kebatasan Darat | Sabah, Kudat | 1981 | 6°42'37.0"N 117°05'07.5"E |
|  | Kg Kapitanagan | Sabah, Bangi Island | 1984 | 7°16'01.9"N 117°08'59.8"E |
|  | Pinanwantai | Sabah, Ranau | 1996-1997 | 6°09'49.3"N 116°46'10.7"E |
|  | Pahu | Sabah, Ranau | 1996-1997 | 5°57'05.0"N 116°32'27.3"E |
|  | Togop | Sabah, Ranau | 1996-1997 | 6°04'01.8"N 116°46'37.5"E |
|  | Tarawas | Sabah, Ranau | 1996-1997 | 6°06'59.2"N 116°48'34.7"E |
|  | Tibabar | Sabah, Ranau | 1996-1997 | 6°10'02.0"N 116°51'24.6"E |
|  | Tumbalang | Sabah, Ranau | 1996-1997 | 6°10'02.2"N 116°51'24.9"E |
|  | Alintang Village | Sabah, Kinabatangan | 2001-2003 | 5°08'54.2"N 116°44'24.8"E |
|  | Mengkawago Village | Sabah, Kinabatangan | 2001-2003 | 5°14'27.7"N 116°48'05.4"E |
|  | Minusu Village | Sabah, Kinabatangan | 2001-2003 | 5°20'48.5"N 116°51'16.3"E |
|  | Saguan Village | Sabah, Kinabatangan | 2001-2003 | 5°22'41.6"N 117°44'30.5"E |
|  | Paradason Village | Sabah, Kudat | 2013 | 6°46′11.75″N, 116°47′3.23″E |
|  | Paradason Village | Sabah, Kudat | 2013-2014 | 6°768’37”N,116°786’35”E |
|  | Timbang Dayang | Sabah, Banggi Island | 2013-2014 | 7°155’85”N,117°102’92”E |
|  | Limbuak laut | Sabah, Banggi Island | 2013-2014 | 7°215’84”N,117°065’75”E |
|  | Tajau laut | Sabah, Kudat | 2013-2014 | 6°57′44.5″N,116°48′56.3″E |
|  | Danum Valley Field Centre | Sabah, Tawau | 2014 | 4°58′N,117°42′E |
|  | Benta Wawasan oil palm plantation | Sabah,Tawau | 2014 | 4°40′N,117°32′E |
|  | Benta Wawasan oil palm plantation | Sabah,Tawau | 2014 | 4°41′N,117°34′E |
|  | Kg. Lau San | Sabah,Kudat | 2014 | 6°54'23.2"N 116°49'38.1"E |
|  | Kg. Tai Cheong | Sabah,Kudat | 2014 | 6°54'52.5"N 116°46'56.5"E |
|  | Kg. Pomonsukan | Sabah,Kudat | 2014 | 6°45'07.2"N 116°45'58.7"E |
|  | Kg. Radtak | Sabah,Kudat | 2014 | 6°44'33.7"N 116°45'25.9"E |
|  | Kg. Nangka | Sabah,Kudat | 2014 | 6°46'59.7"N 116°47'33.4"E |
|  | Kpg. Lajung Kobibi | Sabah,Kudat | 2014 | 6°50'25.8"N 116°41'10.0"E |
|  | Kg. Kindangan | Sabah,Kudat | 2014 | 6°43'03.0"N 116°47'24.4"E |
|  | Kg. Jagil | Sabah,Kudat | 2014 | 6°46'04.1"N 116°39'39.1"E |
|  | Kg. Pinturu | Sabah,Kudat | 2014 | 6°48'42.2"N 116°45'50.6"E |
|  | Kg. Rita | Sabah,Kudat | 2014 | 6°43'01.0"N 116°39'57.6"E |
|  | Kg. Minikodong | Sabah,Kudat | 2014 | 6°44'23.5"N 116°40'40.9"E |
|  | Kg. Pinturu | Sabah,Kudat | 2014 | 6°48'34.1"N 116°45'46.6"E |
|  | Kg. Lodung | Sabah,Kudat | 2014 | 6°49'46.2"N 116°47'06.6"E |
|  | Kg. Lodung | Sabah,Kudat | 2014 | 6°49'20.6"N 116°47'12.1"E |
|  | Kg. Tomohon | Sabah,Kudat | 2014 | 6°48'33.1"N 116°47'27.5"E |
|  | Kg. Narandang | Sabah,Kudat | 2014 | 6°46'58.7"N 116°44'48.3"E |
|  | Kg. Jagil | Sabah,Kudat | 2014 | 6°46'12.9"N 116°39'47.2"E |
|  | Kg. Suang Pa | Sabah,Kudat | 2014 | 6°59'40.3"N 116°47'23.2"E |
|  | Kg. Rondoman | Sabah,Kudat | 2014 | 6°46'41.7"N 116°40'33.2"E |
|  | Kg. Togumamal Laut | Sabah,Kudat | 2014 | 6°45'42.2"N 116°50'38.1"E |
|  | Kg. Kondopi | Sabah,Kudat | 2014 | 6°41'49.5"N 116°47'10.3"E |
|  | Kg. Narandang | Sabah,Kudat | 2014 | 6°46'47.2"N 116°45'05.5"E |
|  | Kg. Mambatu Laut | Sabah,Kudat | 2014 | 6°44'19.7"N 116°48'42.0"E |
|  | Kg. Tinukadan Laut | Sabah,Kudat | 2014 | 6°45'33.5"N 116°49'03.1"E |
|  | Kg. Pomonsukan | Sabah,Kudat | 2014 | 6°45'11.1"N 116°45'58.9"E |
|  | Kg. Paradason B | Sabah,Kudat | 2014 | 6°46'08.9"N 116°48'02.4"E |
|  | Kg. Longgom Besar | Sabah,Kudat | 2014 | 6°57'43.4"N 116°45'02.7"E |
|  | Kg. Kodiung Darat | Sabah,Kudat | 2014 | 6°42'32.2"N 116°39'14.4"E |
|  | Pomunsukan | Sabah,Kudat | 2014-2015 | 6°45.227N’ 116° 45.948E’ |
|  | Tamalang | Sabah,Kudat | 2014-2015 | 6°54’23.6N’116°49’36.8E’ |
|  | Gumandang | Sabah,Kudat | 2014-2015 | 6°58’32.8N’116° 47’11.6E’ |
|  | Timug | Sabah,Kudat | 2014-2015 | 6º43.822N’116° 46.038E’ |
|  | Lotong | Sabah,Kudat | 2014-2015 | 6°44.905N’ 116° 48.018E’ |
|  | Tajau Darat | Sabah,Kudat | 2014-2015 | 6º56.698N’ 116º 46.910E’ |
|  | Kg Onduon | Sabah,Kudat | 2014-2015 | 6° 42.893N’ 116° 47.835E’ |
|  | Paus | Sabah, Ranau | 2015-2016 | 5°41′N,116°47′E |
|  | Keritan Ulu | Sabah, Keningau | 2015-2016 | 5°19′N,116°2′E |
|  | Long Luping | Sarawak, Lawas | 2015 | 4° 16′ 18′′ N, 115° 31′ 49′′ E |
|  | Kem Kayu Balak Merarap | Sarawak, Lawas | 2020 | 4°21'09.9"N 115°27'36.5"E |
|  | Kg. Pundut | Sarawak, Limbang | 2020 | 4°37'39.1"N 114°57'06.1"E |
|  | Rumah Kebun, Ldg Kelapa Sawit Ulu Jelapang | Sarawak, Bintulu | 2020 | 2°43'27.7"N 113°20'57.2"E |
|  | Village | Sabah, Ranau | 2017 | 05º58’N,116º42E |
|  | Simpang Utong | Sarawak, Kapit | 2020 | 2°00'04.0"N 112°58'21.2"E |
|  | Kg. Ulu Sugiang | Sabah, Tenom | 2018-2019 | 4°55'02.1"N 115°53'10.0"E |
|  | Kg. Bt Apar | Sabah, Tenom | 2018-2019 | 5°12'15.9"N 115°57'05.9"E |
|  | Blok 6 LS | Sabah, Tenom | 2018-2019 | 5°12'14.2"N 116°01'09.1"E |
|  | Blok 8 LS | Sabah, Tenom | 2018-2019 | 5°12'59.2"N 116°00'59.0"E |
|  | Kg. Mosolog | Sabah, Tenom | 2018-2019 | 5°15'12.6"N 116°00'45.0"E |
|  | Kg. Tilis | Sabah, Tenom | 2018-2019 | 4°46'29.8"N 115°54'04.0"E |
|  | Kg Kelanaan | Sabah, Tenom | 2018-2019 | 5°11'56.0"N 116°02'25.7"E |
| *An. hackeri* | Pulau Aur | Mersing, Johor | 1965 | 2°35'00.0"N 104°09'52.1"E |
|  | Rantau Panjang | Selangor, Klang | 1961 | 3°04'00.0"N 101°25'00.0"E |
|  | Mangrove Coastal Zone | Selangor, Kuala Selangor |  | 3°20'19.8"N 101°14'41.0"E |
|  | Malay Kampung | Selangor, Rantau Panjang |  | 6.0116°N, 101.9784° E |
|  | Kg. Sg Semungkis | Selangor, Hulu Langat | 2021 | 3°10'34.6"N 101°49'34.0"E |
|  | Hutan Lipur Sungai Batangsi | Selangor, Seminyih | 2020 | 3°02'50.2"N 101°55'36.4"E |
| *An. macarthuri* | Bukit Kertau | Pahang, Chenor | 1970 | 3°04'05.0"N 102°05'59.1"E |
|  | Forest | Sarawak, Kapit | 2005-2006 | 1°56'43.4"N 112°50'09.7"E |
|  | Pos Lenjang | Pahang, Kuala Lipis | 2009 | 4°15.413N' 101°32.843E’ |
|  | - | Sabah, Tawau | 2013-2014 | 4°35'51.9"N 117°35'05.8"E |
|  | Danum Valley Field Centre | Sabah, Tawau | 2014 | 4°58′N,117°42′E |
|  | Benta Wawasan oil palm plantation | Sabah, Tawau | 2014 | 4°40′N,117°32′E |
|  | Benta Wawasan oil palm plantation | Sabah, Tawau | 2014 | 4°41′N,117°34′E |
| *An. pujutensis* | Pacific tin | Selangor, Kuala selangor | 1965 | 3°22'47.0"N 101°41'52.1"E |
|  | Mangrove Coastal Zone | Selangor, Kuala Selangor | 1963 | 3°20'19.8"N 101°14'41.0"E |
|  | Rantau Panjang | Selangor, Klang | 1961 | 3°04'00.0"N 101°25'00.0"E |
|  | Malay Kampung | Selangor | 1962 | 6.0116°N, 101.9784° E |
|  | Bukit Kertau | Pahang, Chenor | 1970 | 3°04'05.0"N 102°05'59.1"E |
|  | Serunai Mela | Pahang, Kuala Lipis | 2007-2008 | 4° 7.0'N, 102° 11.9'E |
|  | Sg. Ular | Pahang, Kuala Lipis | 2007-2008 | 4° 15.7'N, 102°4.8'E |
|  | Paus | Sabah, Ranau | 2015-2019 | 5°41′N, 116°47′E |
|  | Serunai Mela | Pahang, Kuala Lipis | 2017 | 4° 7.0'N, 102° 11.9'E |
|  | Forest | Sarawak, Kapit | 2005-2006 | 1°56'43.4"N 112°50'09.7"E |
