# Supplementary material for: Spatial analyses of Plasmodium knowlesi vectors with reference to control interventions in Malaysia
Source: Parasit Vectors. 2023 Oct 9;16:355. doi: 10.1186/s13071-023-05984-x (PMC10563288; doi:10.1186/s13071-023-05984-x)

**Additional file 2.**

**Fig. S1: Maps show the elevation, forest cover, forest loss, temperature and water bodies of Malaysia.**


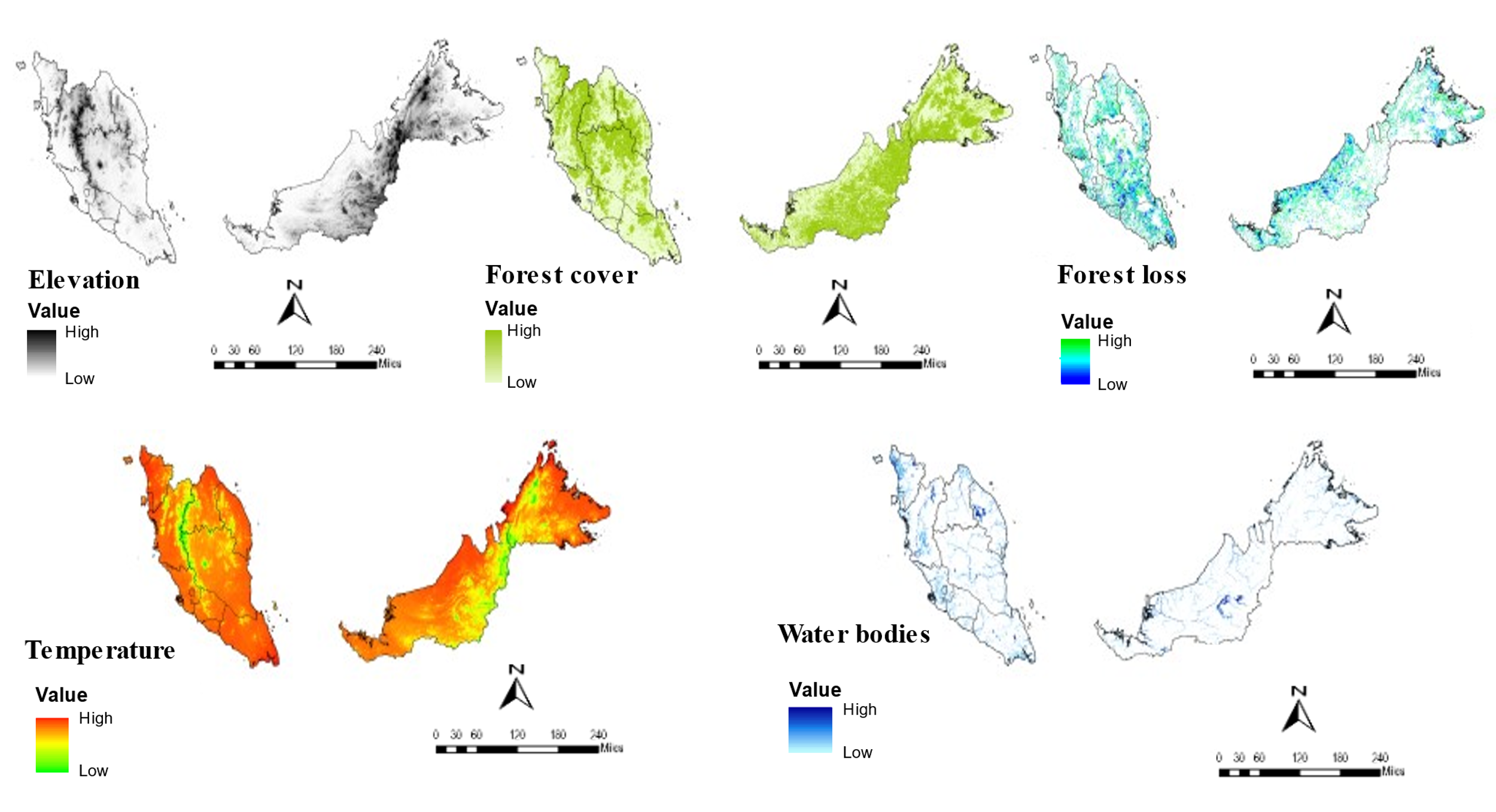

Supplement: Supplementary file 2 — Additional file 2: Figure S1. Maps showing the elevation, forest cover, forest loss, temperature and water bodies of Malaysia. [file 13071_2023_5984_MOESM2_ESM.docx]
